# Dimeric 3,5-Bis(benzylidene)-4-piperidones: Tumor-Selective Cytotoxicity and Structure-Activity Relationships

**DOI:** 10.3390/medicines11010003

**Published:** 2024-01-11

**Authors:** Swagatika Das, Praveen K. Roayapalley, Hiroshi Sakagami, Naoki Umemura, Dennis K. J. Gorecki, Mohammad Hossain, Masami Kawase, Umashankar Das, Jonathan R. Dimmock

**Affiliations:** 1 Drug Discovery and Development Research Cluster, University of Saskatchewan, Saskatoon, SK S7N 5E5, Canadajr.dimmock@usask.ca (J.R.D.); 2 Meikai University Research Institute of Odontology, Saitama 350-0283, Japan; sakagami@dent.meikai.ac.jp; 3 Department of Oral Biochemistry, Asahi University School of Dentistry, Gifu 501-0296, Japan; umemura@dent.asahi-u.ac.jp; 4 School of Sciences, Indiana University Kokomo, Kokomo, IN 46904-9003, USA; mohoss@iu.edu; 5 Faculty of Pharmaceutical Sciences, Matsuyama University, Matsuyama 790-8578, Japan; kawase@g.matsuyama-u.ac.jp

**Keywords:** unsaturated ketones, cytotoxicity, selective toxicity, QSAR, modes of action

## Abstract

***Background:*** The objective of this study is to find novel antineoplastic agents that display greater toxicity to malignant cells than to neoplasms. In addition, the mechanisms of action of representative compounds are sought. This report describes the cytotoxicity of a number of dimers of 3,5-bis(benzylidene)-4-piperidones against human malignant cells (promyelocytic leukemia HL-60 and squamous cell carcinoma HSC-2, HSC-3, and HSC-4). ***Methods:*** Tumor specificity was evaluated by the selectivity index (SI), that is the ratio of the mean CC_50_ for human non-malignant oral cells (gingival fibroblasts, pulp cells, periodontal ligament fibroblasts) to that for malignant cells. ***Results:*** The compounds were highly toxic to human malignant cells. On the other hand, these molecules were less toxic to human non-malignant cells. In particular, a potent lead molecule, **3b**, was identified. A QSAR study revealed that the placement of electron-releasing and hydrophilic substituents into the aryl rings led to increases in cytotoxic potencies. The modes of action of a lead compound discovered in this study designated **3b** were the activation of caspases-3 and -7, as well as causing PARP1 cleavage and G2 arrest, followed by sub-G1 accumulation in the cell cycle. This compound also depolarized the mitochondrial membrane and generated reactive oxygen species in human colon carcinoma HCT116 cells. In conclusion, this study has revealed that, in general, the compounds described in this report are tumor-selective cytotoxins.

## 1. Introduction

**Figure 1:**
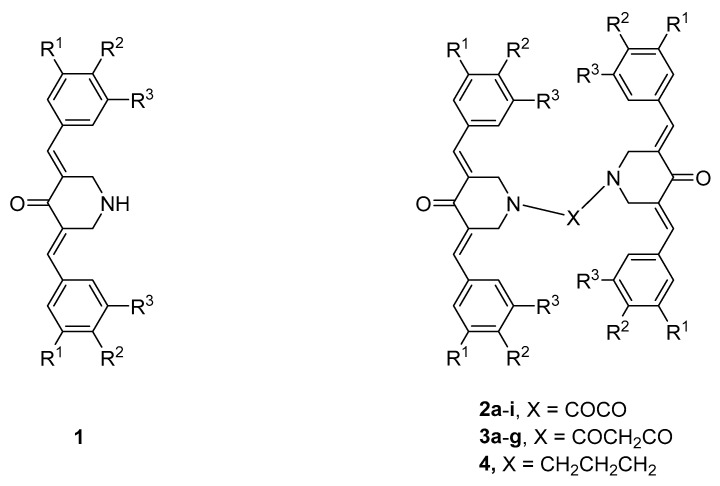
Structures of the compounds in Series **1**–**4**. The aryl substituents in Series **2** and **3** are as follows: **a**: R^1^ = R^2^ = R^3^ = H; **b**: R^1^ = R^3^ = H, R^2^ = F; **c**: R^1^ = R^3^ = H, R^2^ = Cl; **d**: R^1^ = R^2^ = Cl, R^3^ = H; **e**: R^1^ = R^3^ = H, R^2^ = CH_3_; **f**: R^1^ = R^2^ = (OCH_3_)_2_, R^3^ = H; **g**: R^1^ = R^2^ = R^3^ = OCH_3_; **h**: R^1^ = R^3^ = H, R^2^ = OCH_3_; **i**: R^1^ = R^3^ = H, R^2^ = N (CH_3_)_2_. In the case of Compound **4**, R^1^ = R^2^ = R^3^ = H.

A major interest in our laboratories is the synthesis of novel α,β-unsaturated ketones, which were designed as candidate antineoplastic agents [1]. A number of these compounds react with thiols [2,3,4], which is likely an important contributor to cytotoxic effects. In particular, we are interested in 3,5-bis(benzylidene)-4-piperidones, whose general structure is displayed in Figure 1. However, the aim of the present investigation is not only to find compounds with marked antineoplastic properties but those that display greater toxicity to neoplasms than to non-malignant cells. This selective toxicity may arise since after an initial chemical insult, cancer cells may be more susceptible or more sensitive to subsequent toxic effects than non-malignant cells are [5,6]. Thus, in Series **2**–**4**, whose structures are displayed in Figure 1, there are four sites at which sequential interactions with cellular constituents could occur.

Previous studies with dimeric compounds are as follows [7]. The initial study used a diacyl linker and the compounds displayed potent cytotoxicity towards a variety of leukemic cells and lymphomas [7]. A subsequent study revealed that these compounds were cytotoxic to colon cancers, as well as leukemias [8]. An important observation was the discovery of the multidrug-resistant revertant properties of these compounds [8]. Subsequently, Series **2**–**4** were prepared and most of these compounds demonstrated high potency to various colon cancers, as well as leukemic and lymphoma cells [9]. However, limited evidence was provided as to whether greater toxicity for neoplastic cells was being demonstrated or not. Hence, the decision was made to evaluate the compounds in Series **2**–**4** against the HL-60, HSC-2, HSC-3, and HSC-4 neoplastic cell lines, as well as the HGF, HPC, and HPLF non-malignant cells. Should significant potency and especially selective toxicity be demonstrated, QSAR and mode of action investigations would be envisaged.

## 2. Materials and Methods

### 2.1. Syntheses of Compounds

The synthesis of the compounds has been described previously [7].

### 2.2. Cytotoxicity Assays

Human promyelocytic leukemia [HL-60 (RCB3683)] (purchased from Riken Cell Bank, Tsukuba, Japan) was cultured at 37 °C in RPMI1640 medium (Fujifilm Wako Pure Chemical Co., Osaka, Japan) supplemented with 10% heat (56 °C, 30 min) inactivated FBS (Sigma-Aldrich Inc. St. Louis, MO, USA), 100 U/mL penicillin G, and 100 µg/mL streptomycin sulfate in a humidified 5% CO_2_ incubator (MCO-170 AICUVD-P, Panasonic Healthcare Co., Ltd., Gunma, Japan). Human oral squamous cell carcinoma (OSCC) cell lines derived from tongue [HSC-2 (RCB1945), HSC-3 (RCB1975), HSC-4 (RCB1902)] (Riken Cell Bank), and human normal oral cells (gingival fibroblasts, HGF; pulp cell, HPC; periodontal ligament fibroblast, HPLF), established from the first premolar extracted tooth in the lower jaw and periodontal tissues of a twelve-year-old girl, according to the guidelines of Meikai University Ethic Committee (No. A0808), were cultured in DMEM medium (Fujifilm Wako Pure Chemical Co.) supplemented with 10% FBS and antibiotics. The compounds in Series **2**–**4** were evaluated against HL-60, HSC-2, HSC-3, HSC-4, HGF, HPC, and HPLF cells based on a literature procedure, as described previously [1]. In brief, near confluent cells were incubated in triplicate for 48 h at 37 °C with different concentrations (0, 0.039, 0.078, 0.156, 0.312, 0.625, 1.25, 2.5, 6.3, 12.5, 25, 50, 100 µM) of the compounds; then, viable cell numbers were measured using the MTT method. From the dose–response curve, a 50% cytotoxic concentration (CC_50_) was determined. All samples were dissolved in dimethylsulfoxide (DMSO). The toxicity of DMSO alone was calculated and subtracted. The CC_50_ values were determined from dose–response curves.

### 2.3. Activation of Caspases-3 and -7

Both **3b** (0.5 µM) and **3c** (0.5 µM) were examined for their ability to activate caspases-3 and -7 in HSC cells. This determination was accomplished using the Caspase-Glo 3/7 assay (Promega Corporation, Madison, WI, USA). In brief, this assay uses a tetrapeptide sequence DEVD, which is optimized for caspase and luciferase activities. The cell lysate was incubated with the Caspase-Glo reagent (buffer and substrate) for 1 h and the luminescence was measured. The resultant luminescent signal produced by luciferase was confirmed to be proportional to the amount of caspase-3 and -7 activity.

### 2.4. PARP1 Cleavage and Activation of Bim, Bax, Puma, and Bak

Antibodies against cleaved PARP1, bim, bax, puma, and bak were obtained from Cell Signaling Technology (Beverley, MA, USA) while the anti-actin antibody was purchased from Santa Cruz Biotechnology (Santa Cruz, CA, USA).

The HSC-2 cells were cultured for 24 h and, then, treated with 0.5 µM of **3b** or **3c** for 24 h. The cells were scraped and collected in lysis buffer (purchased from Cell Signaling Technology), which contained 1 mM of phenylmethanesulfonyl fluoride and one tablet of protease inhibitor cocktail (Complete, EDTA-free; Roche Diagnostics, Mannheim, Germany). The proteins were subjected to 8% SDS-polyacrylamide gel electrophoresis and, then, transferred to a polyvinylidene difluoride membrane saturated with 5% nonfat dry milk. The membranes were incubated with rabbit monoclonal anti-cleaved PARP (Cell Signaling Technology, Beverly, MA, USA; #9541, 1:1000), or beta-actin antibody (Sigma-Aldrich, St Louis, MO, USA; A5441, 1:10,000) for 1 h at room temperature. The membranes were washed with Tris-buffered saline containing 0.1% Tween 80. And, the secondary antibodies were horseradish peroxidase-conjugated goat antimouse or antirabbit, which reacted with the chemoluminescent substrate system and were exposed to X-ray films.

### 2.5. Cell Cycle Analysis

The effect of **3b** (0.5 µM) and **3c** (0.5 µM) on the phase distribution of HSC-2 cells after 24 h of incubation was determined using the protocol of a manufacturer (BD Pharmingen, BD BioSciences, San Jose, CA, USA). In brief, treated and untreated cells were suspended at a density of ~1 × 10^6^ cells/mL in cooled phosphate-buffered saline (PBS) without calcium and magnesium ions and fixed with 70% ethanol overnight at −20 °C. The cells were washed with cooled PBS and re-suspended in a propidium iodide/RNase staining buffer in the dark at room temperature for 0.25 h. Both acquisition and analysis were performed using EPICS ALTRA (Beckman Coulter, Brea, CA, USA) and EXPO 32 software.

### 2.6. Mitochondrial Membrane Potential Determination

The determination of the IC_50_ values of **3b**, **3c**, and 5-FU on the mitochondrial membrane potential in HCT 116 cells was undertaken using a literature procedure [10]. In brief, the cells were incubated at 37 °C in an atmosphere of 5% carbon dioxide for 24 h. The compounds were dissolved in DMSO, added to the cells, and incubated for 48 h. Then, a fluorescent dye, *m*-chlorophenylhydrazone (CCCP), and 2,4-dinitrophenol (DNP) were added to the cells, which were incubated for 0.5 h. After the addition of tetramethylrhodamine ethyl ester, the fluorescence of the cells was determined using a plate reader.

### 2.7. Reactive Oxygen Species Determination

The determination of the effects of **3b**, **3c**, and 5-FU on the generation of reactive oxygen species in HCT 116 cells was accomplished using a literature procedure [11]. In brief, the cells were incubated at 37 °C in an atmosphere of 5% carbon dioxide for 24 h. Subsequently, solutions of the compounds in DMSO were added to the cells, which were incubated for 48 h. After 2′,7′-dichlorofluorescein was added to the cells, incubation continued for another 0.5 h and the fluorescence of the cells was measured spectrophotometrically (excitation wavelength 485 nm; emission wavelength, 530 nm).

### 2.8. Quantitative Structure-Activity Relationships

The physicochemical constants for the aryl substituents in **2a**–**g** and **3a**–**g**, which were used in the QSAR determinations, were taken from the literature [12]. The linear and semilogarithmic plots were made using a commercial statistical package [13].

## 3. Results

The compounds in Series **2** and **3** were prepared by reacting an amount of 3,5-bis(benzylidene)-4-piperidones **1** with oxalyl chloride to produce **2a**–**i** or with malonyl chloride to form **3a**–**g**. In addition, a related compound, Compound **4**, was prepared by reacting **1a** (**1**, R^1^ = R^2^ = R^3^ = H) with 1,3-dibromopropane. These unsaturated ketones were evaluated against human promyelocytic leukemia cells (HL-60), as well as the human oral squamous cell carcinoma cell lines HSC-2, HSC-3, and HSC-4. These data are presented in Table 1 and reveal that, in general, the compounds in Series **2**–**4** are potent cytotoxins, which are much more toxic than the two reference compounds melphalan and curcumin. The compounds displaying the greatest average cytotoxic potencies are **2a**, **b**, **g**, **3a**, **b**, and **g**. In addition, these compounds were evaluated against human gingival fibroblasts (HGFs), human pulp cells (HPCs), and human periodontal ligament fibroblasts (HPLFs), which are non-malignant cells. These results are displayed in Table 2. Compounds with the highest CC_50_ values, i.e., those that are best tolerated by the non-malignant cells with average CC_50_ values in excess of 50 µM, are **2c**, **h**, **i**, and **3d**, as well as the drug melphalan. Selectivity index (SI) figures were generated by dividing the average CC_50_ values towards HGF, HPC, and HPLF cells by the CC_50_ value towards a neoplastic cell line. These results are displayed in Table 1. The compounds with the highest SI values are **2f** and **3b**, which are clearly lead molecules. A QSAR study revealed that cytotoxic potencies were correlated positively with the magnitude of the Hammett sigma (σ) values and Hansch pi (π) values of the aryl substituents. In addition, the ADME properties of seven potent cytotoxins were investigated and the result is presented in Table 3. The results reveal that future studies with analogs of Series **2**–**4** should have lower molecular weights and hydrophobicity than the current analogs.

**Figure 2:**
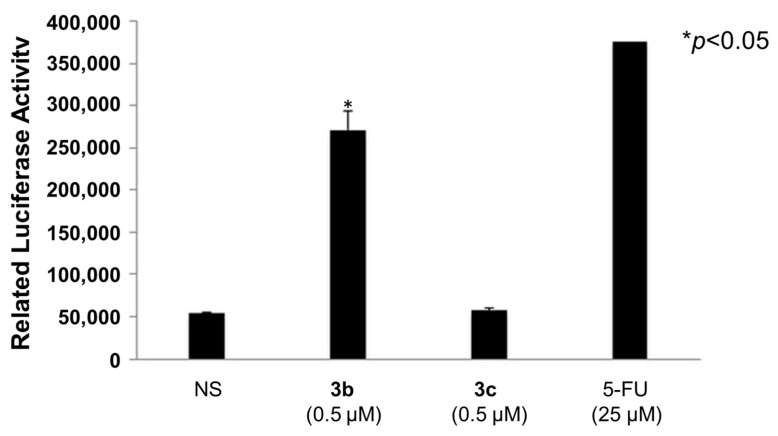
Evaluation of **3b** and **3c** for the induction of caspases-3 and -7 in HSC-2 cells; 5-Fluorouracil (5-FU, 25 µM) was used as a positive control. Each value represents mean ± S.D. (*n* = 3) * *p* < 0.05 (statistically significant from non-stimulation, NS). The CC_50_ values of **3b** and **3c** in HSC-2 cells were 0.041 and 5.5 µM (two orders of difference); the concentration of 0.5 µM (in between) was selected for this experiment.

**Figure 3:**
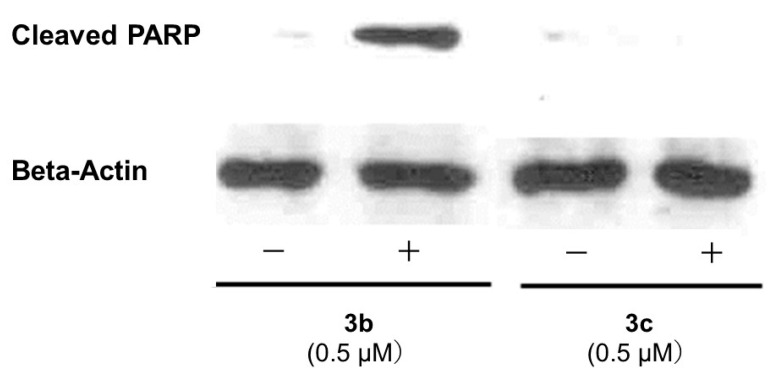
Examination of **3b** (0.5 µM) and **3c** (0.5 µM) for the cleavage of PARP1 in HSC-2 cells.

**Figure 4:**
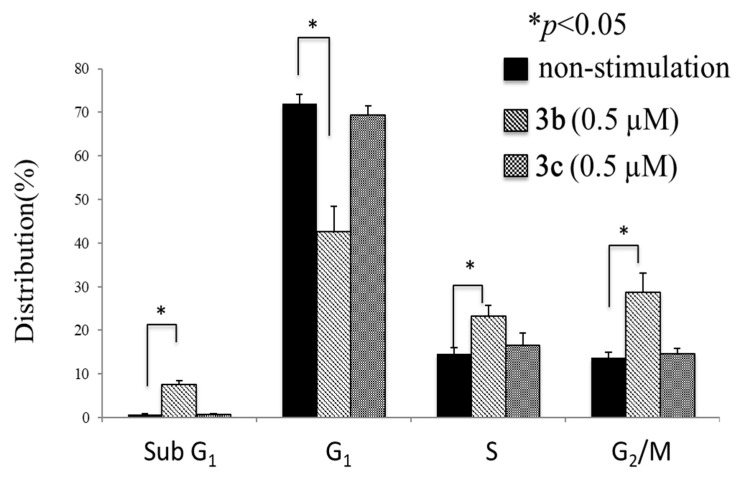
Cell cycle analysis of HSC-2 cells after treatment with **3b** (0.5 µM) and **3c** (0.5 µM). Each value represents mean ± S.D. (*n* = 3) * *p* < 0.05 (statistically significant from non-stimulation, NS).

**Figure 5:**
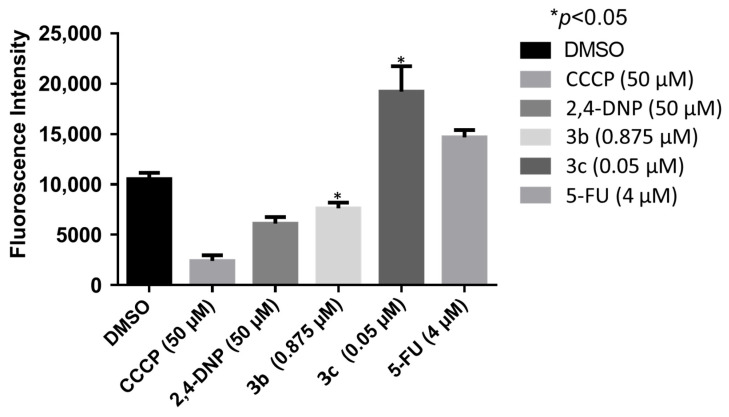
The effect of **3b** and **3c** on the mitochondrial membrane potential of HCT116 cells. Each value represents mean ± S.D. (*n* = 3) * *p* < 0.05 (statistically significant from control, DMSO).

**Figure 6:**
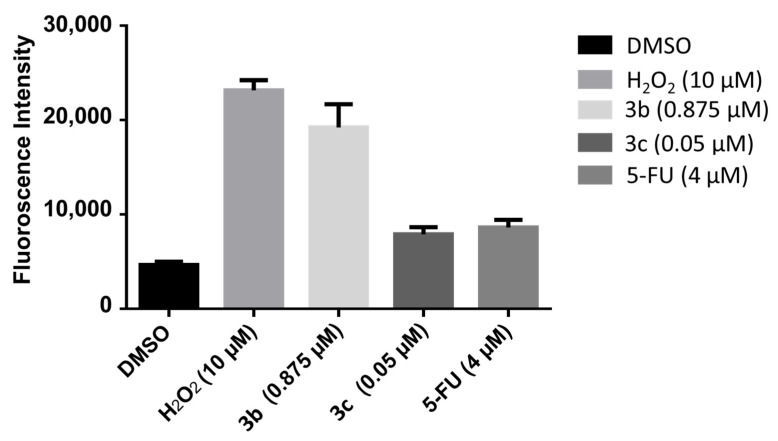
The efficacy of **3b** and **3c** in generating reactive oxygen species in HCT116 cells. Each value represents mean ± S.D. (*n* = 3), *p* < 0.05 (statistically significant from control, DMSO).

A number of mode of action studies were undertaken with **3b** and **3c** using HSC-2 cells. The enone **3b** induced caspases-3 and -7 (Figure 2), causing the cleavage of PARP (Figure 3) and G2M arrest in the cell cycle (Figure 4). Both compounds were evaluated for their effect on the mitochondrial membrane potential (Figure 5) and generating reactive oxygen species in HCT116 cells (Figure 6).

## 4. Discussion

The results of evaluating **2a**–**i**, **3a**–**g**, and **4** against the HL60, HSC-2, HSC-3, and HSC-4 neoplastic cells are presented in Table 1. With the exception of **2i** and **3d**, the compounds are potent cytotoxins. No less than 53% of the CC_50_ values are submicromolar and, if one removes the data for the two outliers **2i** and **3d**, then, 60% of the CC_50_ values are submicromolar. Of particular note are five compounds that have CC_50_ values in the double-digit nanomolar range (10^−8^ M) in all four bioassays, namely, **2b** (4-F), **2g** [3,4,5-(OCH_3_)_3_], **3a** (H), **3b** (4-F), and **3g** [3,4,5-(OCH_3_)_3_], and are lead molecules.

The importance of the spacer group between the two piperidyl nitrogen atoms was addressed. The outliers **2i** and **3d** (as well as **2d**) were omitted from consideration. The average CC_50_ values of **2a**–**c**, **e**–**g** and **3a**–**c**, **e**–**g** are 1.62 and 1.57 µM, respectively, indicating that both linker groups may be used in developing these compounds. The average CC_50_ values of the three carbon spacers in **3a** and **4** are 0.06 and 0.21 µM, respectively, revealing that the two oxygen atoms in the malonyl group of **3a** enhance cytotoxic potencies in general.

Comparisons were made between the potencies of the compounds in Series **2**–**4** and established antineoplastic agents. Melphalan was chosen as a reference compound since, like conjugated enones, it is an alkylating agent. This drug has good potency towards HL-60 cells. However, it has low toxicity towards HSC-2, HSC-3, and HSC-4 cells and, thus, virtually all of the compounds in Series **2**–**4** are more potent than melphalan. Curcumin was selected since it is an enone and has antineoplastic properties [14]. However, in general, the biodata of Series **2**–**4** in Table 1 and Table 2 are markedly superior to those displayed by curcumin in terms of antineoplastic properties and SI values.

While it is important to find compounds that will kill neoplastic cells, the identification of molecules that have a substantially greater toxicity to neoplasms than to non-malignant cells is a major objective of this study. Hence the compounds in Series **2**–**4** were evaluated against HGF, HPC, and HPLF non-malignant cells. These data are presented in Table 2.

The data in Table 2 reveal considerable variations in the average CC_50_ values, which range from 0.19 µM (**3g**) to >100 µM (**2i**, **3d**). Of primary interest are the compounds with high CC_50_ values (less toxic compounds). In this regard, compounds with average CC_50_ values of greater than 10 µM are **2c**–**f**, **h**, and **i** and **3c**–**e**, i.e., a little over half of the compounds. Particularly noteworthy are the average CC_50_ results for both **2c**, **d**, and **e** and **3c**, **d**, and **e**, which reveal that compounds with the 4-chloro-3,4-dichloro and 4-methyl aryl substituents are found in compounds with low cytotoxicity. One should note the tolerance of HGF, HPC, and HPLF cells to the reference compounds melphalan and curcumin.

Under clinical conditions, tumors are surrounded by non-malignant cells. Thus, in order to examine whether greater toxicity was displayed by the tumors than by normal cells, selectivity index (SI) figures were computed. These SI values were obtained by dividing the average CC_50_ values towards HGF, HPC, and HPLF non-malignant cells by the CC_50_ figures towards a specific cancer cell line. The SI values are presented in Table 1. The following observations may be made. Virtually all of the SI values recorded in Table 1 are greater than 1, providing evidence of the ability of the compounds in Series **2**–**4** to display tumor-selective toxicity. The average SI values of the compounds in Series **2**–**4** are greater than 10 in the case of **2a**–**c**, **e**, **f**, and **g**, as well as in **3a**, **b**, **e**, and **4**, i.e., in 59% of the cases. It is of interest to note that there are twice as many compounds in Series **2** than those that are found in Series **3**, which have SI values greater than 10. In particular, the average SI figures for **2f** (57.0) and **3b** (133) identify them as being important lead molecules.

In order to obtain some guidelines regarding expanding the project in the future, a quantitative structure-activity relationship (QSAR) approach was undertaken in regard to both potency and selectivity. In the case of potency, the electronic, hydrophilic, and steric properties of the aryl substituents in **2a**–**g** and **3a**–**g** are influenced by the magnitude of the Hammett σ, Hansch π, and molar refractivity (MR) constants of the aryl substituents. Linear plots were made between the CC_50_ values of **2a**–**g** in the HL-60 cells and, first, the σ values, then, the π constants, and, subsequently, the MR values.

This process was repeated using the CC_50_ figures of **2a**–**g** towards HSC-2, HSC-3, and HSC-4 cells. Then these determinations were repeated, except the CC_50_ values of **3a**–**g**, not **2a**–**g**, were used. All of these determinations were repeated, except semilogarithmic plots instead of linear ones were made.

The results obtained are presented in Tables S1 (data of **2a**–**g**) and S2 (results of **3a**–**g**) in the Supplemental section of this report. The following observations and conclusions were drawn:

(1) A statistically significant positive relationship between the CC_50_ figures of **2a**–**g** and the sigma (σ) values was obtained in a little over half of the observations made. This result reveals that the electronic properties of the aryl substituents play a significant albeit minor role in determining the magnitude of the CC_50_ values. The correlation is positive, revealing that the CC_50_ values rise (potency diminishes) as the sigma (σ) values increase in magnitude;
(2) The data in Table S1 reveal clearly the positive correlation between the CC_50_ values and the π constants. Thus, potency increases as the hydrophilicity of the molecules falls;
(3) There is no correlation between the CC_50_ values of **2a**–**g** and the MR values of the aryl substituents;
(4) The results in Table S2 for **3a**–**g** reveal a similar pattern as displayed by **2a**–**g**. There is a statistically significant positive correlation between the sigma (σ) values and the CC_50_ data in half of the determinations. The importance of the π values, but not the MR constants, in controlling potencies is clearly demonstrated. Thus, in planning the expansion in this series of compounds, groups should be placed in the aryl rings, which are either electron-donating, hydrophilic, or have both of these properties. Examples of such substituents are the 4-hydroxy, 4-amino, and 4-methoxy substituents with sigma (σ) values of −0.37, −0.66, and −0.27, respectively ([12], p. 49). Regarding the hydrophilic groups, the cyano, carboxylic acid, and methylsulfonyloxy have π values of −0.57, −0.32, and −0.88, respectively ([12], p. 49).

A further issue to consider is whether the magnitude of the selective toxicity is influenced by the electronic, hydrophobic, and steric properties of the groups in the aryl rings. Consequently, linear and semilogarithmic plots were made between the average SI values of **2a**–**g** and the σ, π, and MR constants in the aryl rings of these compounds. A similar procedure was undertaken with **3a**–**c**, **e**–**g** (**3d** does not have a specific average SI figure). The results of this analysis are presented in Supplementary Table S3. In the case of Series **2**, the semilogarithmic plots revealed a negative correlation between the σ and π values. No other correlations were noted (*p* > 0.05).

Two highly important features of a candidate antineoplastic agent are their potency and SI value. In order to find lead compounds that combine both of these properties, potency-selectivity expression (PSE) figures for the compounds in Series **2**–**4** were obtained. The values are the products of the reciprocal of the average CC_50_ value against neoplastic cells and the average SI figure times 100. These data are presented in Table 2. The following compounds have PSE figures in excess of 100, namely, **2a**, **b**, **f**, **g**, **3a**, **b**, **g**, and **4**. The 4-fluoro analogs **2b** and **3b** have the highest PSE values and are lead molecules. The PSE values of the unsubstituted compounds **3a** and **4** are 290 and 120, respectively, indicating the contribution of the oxygen atoms in the spacer group of **3a** to its greater PSE value than **4**. One may also note that virtually all of the compounds in Series **2**–**4** have PSE figures far higher than those displayed by melphalan and curcumin.

An effort was made to find out if representative compounds in Series **2** and **3** possess drug-like properties. Accordingly, three compounds in both Series **2** and **3**, namely, **2a**, **b**, and **g** and **3a**, **b**, and **g**, which have the same aryl substituents, were considered as they have good PSE figures. The rule of five articulated by Lipinski et al. [15] pertains to the size of the molecular weights, LogP values, and the numbers of hydrogen bond acceptor and donor atoms while Veber et al. [16] suggest that the number of rotatable bonds and the total polar surface (TPSA) are important physicochemical parameters. The results are portrayed in Table 3. In general, the compounds have favorable properties in terms of hydrogen bonding, rotatable bonds, and TPSA. However, the size and hydrophobicity of these molecules suggest that smaller molecules should be created with lower hydrophobic properties. The modes of action of these compounds were addressed, initially in general terms and then with specific targets in mind. As mentioned previously, a number of investigations revealed that conjugated enones react readily with thiols [2,3,4]. Specifically, various enones react only with thiol groups, even when other functionalities, such as amino and hydroxyl groups, are present [17].

Some of the biochemical mechanisms whereby an important lead compound, **3b**, exerts its cytotoxic properties were investigated; also, a question was addressed, namely, why is **3b** more potent than related analogs, such as **3c**? The following investigations were undertaken using HSC-2 cells and the same concentrations of compounds. Caspases-3 and -7 are activated by both the death receptor and mitochondrial pathways, which lead to apoptosis. After 24 h of incubation, **3b**, but not **3c**, induced caspases-3 and -7 and this result is presented in Figure 2. Single-stranded DNA breaks can be repaired by poly(ADP-ribose)polymerase 1 (PARP1) [18] and, thus, compounds that cleave PARP1 reduce the extent of DNA repair and, therefore, may contribute to cytotoxic effects [19]. Figure 3 reveals that **3b**, in contrast to **3c**, leads to the cleavage of PARP1. Cytotoxic agents exert their bioactivity by interfering with the progression of the cell cycle. Cell cycle analysis of **3b** and **3c** reveals that **3b**, but not **3c**, causes a G2/M arrest in HSC-2 cells (Figure 4). The effect of **3b** and **3c** on pro-apoptotic proteins, such as bim, bax, puma, and bak, was examined in HSC-2 cells but no changes in the concentrations of these proteins were observed after 24 h (data not shown). Hence, the way in which **3b** displays its cytotoxic properties includes the activation of caspases -3 and -7, cleavage of PARP1, and G_2_/M arrest, leading to producing a sub-G1 population (DNA fragments produced by activated DNases) [20] in the cell cycle. On the other hand, at the same concentration of 0.5 µM, **3c** does not exert these effects, which explains, at least in part, the greater potency of **3b** than **3c**.

These mode of action studies were conducted using HSC-2 cells. The decision was made to investigate possible ways in which cytotoxicity is mediated using another cell line. Both **3b** and **3c** have been evaluated against human HCT116 colon cancer cells and possess IC_50_ values of 0.60 and 0.05 µM, respectively. One way in which cytotoxicity can be mediated is by lowering the mitochondrial membrane potential (MMP). The data presented in Figure 5 were generated using the mitochondriotropic dye tetramethylrhodamine ethyl ester, which is extruded from the mitochondria into the cytosol when the MMP is lowered. The results presented in Figure 5 reveal that **3b** has a huge effect on lowering the MMP; whereas, **3c** has no effect at the concentrations utilized. Another way in which cytotoxicity can be achieved is by increasing the concentration of reactive oxygen species (ROS) [21]. In this case, the magnitude of the fluorescence of the dye 2′,3′-dichlorofluorescein diacetate revealed that, while **3b** and **3c** cause an increase in the ROS concentrations, **3b** has approximately twice the potency of **3c** (Figure 6). One may conclude that the generation of ROS contributes to the cytotoxicity observed.

In summary, this study has led to the identification of three series of enones, **2**–**4**, which, in general, have far greater toxicity to various neoplasms than non-malignant cells. QSAR studies revealed the importance of the electronic and hydrophobic properties of the aryl substituents. The ADME studies revealed that reducing the size and hydrophobicity of the aryl substituents should be undertaken.

## 5. Conclusions

The results generated in this study reveal that the majority of the compounds in Series **2** and **3** are potent cytotoxins and display greater toxicity to some neoplasms than to various non-malignant cells. From the data presented in Table 1 and Table 2, the compounds **2a**, **b**, **g**, **3a**, **b**, and **g** are lead molecules in terms of potency while **2f** and **3b** have the highest SI values. The PSE values, which take into consideration both potency and SI figures, reveal that the 4-fluoro analogs **2b** and **3b** have the highest PSE values and are lead molecules. A QSAR study indicated that, in order to increase potency, future studies should introduce hydrophilic, electron-donating substituents into the aryl rings. To some extent, this assessment aligns with the ADME result, which suggests that smaller hydrophilic molecules may have preferential absorption characteristics. Various mode of action studies revealed a number of ways the lead molecule **3b** exerts its cytotoxic properties.

From the investigations described in this report, plans may be formulated to expand this project in different ways. For example, the preparation of analogs of **3b** could include the syntheses and cytotoxic evaluation of structural isomers of **3b** and changing the nature and size of the spacer group between the piperidine nitrogen atoms. Further bioevaluations could include assessing **3b** and other lead compounds against other cell lines, both neoplastic and non-malignant, as well as conducting an Ames test. If these results are encouraging, metabolic and pharmacokinetic studies should be implemented.

## Figures and Tables

**Table 1:** Evaluation of the compounds in Series **2**–**4** against human malignant cells.

Compound	Human Tumour Cell Lines, CC_50_ (μM) ^a^									
	HL-60	SI ^b^	HSC-2	SI ^b^	HSC-3	SI ^b^	HSC-4	SI ^b^	AVE. CC_50_	AVE SI ^b^
**2a**	0.11 ± 0.009	29.6	0.085 ± 0.013	38.4	0.15 ± 0.022	21.7	0.20 ± 0.011	16.3	0.14	26.5
**2b**	0.037 ± 0.017	40.5	0.037 ± 0.009	40.5	0.066 ± 0.008	22.7	0.033 ± 0.009	45.5	0.04	37.3
**2c**	4.4 ± 0.17	16.9	3.7 ± 0.60	20.1	11 ± 1.1	6.76	6.7 ± 3.0	11.1	6.45	13.7
**2d**	8.4 ± 0.017	4.08	5.9 ± 0.12	5.81	8.4 ± 0.87	4.08	6.4 ± 0.15	5.36	7.28	4.83
**2e**	2.7 ± 0.78	16.7	1.8 ± 0.25	25.0	4.3 ± 1.1	10.5	2.3 ± 0.25	19.6	2.78	18.0
**2f**	0.19 ± 0.026	65.8	0.42 ± 0.025	29.8	0.23 ± 0.097	54.4	0.16 ± 0.024	78.1	0.25	57.0
**2g**	0.037 ± 0.002	15.4	0.063 ± 0.009	9.05	0.035 ± 0.006	16.3	0.088 ± 0.029	6.48	0.06	11.8
**2h**	15 ± 0.42	>5.45	7.8 ± 1.3	>10.5	9.2 ± 4.7	>8.88	9.6 ± 0.17	>8.51	10.4	>8.34
**2i**	>100	~1.00	>100	~1.00	>100	~1.00	>100	~1.00	>100	~1.00
**3a**	0.046 ± 0.009	20.0	0.050 ± 0.001	18.4	0.046 ± 0.026	20.0	0.084 ± 0.005	11.0	0.06	17.4
**3b**	0.028 ± 0.001	151	0.041 ± 0.004	103	0.046 ± 0.011	92.0	0.023 ± 0.003	184	0.04	133
**3c**	5.0 ± 0.76	8.26	5.5 ± 0.65	7.51	13 ± 2.2	3.18	5.5 ± 0.96	7.51	7.25	6.62
**3d**	65 ± 6.1	>1.54	73 ± 3.5	>1.37	76 ± 13	>1.32	79 ± 5.5	>1.27	73.3	>1.38
**3e**	1.4 ± 0.15	17.6	1.6 ± 0.39	15.4	2.2 ± 0.20	11.2	1.9 ± 0.15	13.0	1.78	14.3
**3f**	0.26 ± 0.021	5.77	0.34 ± 0.076	4.41	0.20 ± 0.14	7.50	0.128 ± 0.014	11.7	0.23	7.35
**3g**	0.030 ± 0.0002	6.33	0.065 ± 0.016	2.92	0.035 ± 0.005	5.43	0.038 ± 0.009	5.00	0.04	4.92
**4**	0.18 ± 0.011	22.2	0.21 ± 0.032	19.1	0.35 ± 0.11	11.4	0.083 ± 0.021	48.2	0.21	25.2
Melphalan	0.89 ± 0.17	75.6	12 ± 2.7	5.61	46 ± 31	1.46	46 ± 6.0	1.46	26.2	21.0
Curcumin	7.8 ± 0.58	5.81	10 ± 0.81	4.53	16 ± 2.7	2.83	14 ± 3.2	3.24	12.0	4.10

^a^ The CC_50_ values are the concentrations of the compounds required to kill 50% of the cells. ^b^ The letters SI refer to the selectivity index. The figures are obtained by dividing the average CC_50_ values towards non-malignant cells (Table 2) by the specific CC_50_ figure towards a specific neoplastic cell line.

**Table 2:** Evaluation of the compounds in Series **2**–**4** against human non-malignant cells.

Compound	Human Normal Cells, CC_50_ (μM) ^a^				PSE ^b^
	HGF	HPC	HPLF	AVE CC_50_	
**2a**	3.8 ± 0.52	0.68 ± 0.073	5.3 ± 0.50	3.26	189
**2b**	1.2 ± 0.10	1.4 ± 0.11	1.9 ± 0.36	1.50	933
**2c**	56 ± 6.7	78 ± 37	89 ± 9.3	74.3	2.12
**2d**	36 ± 3.1	23 ± 3.1	44 ± 3.2	34.3	0.66
**2e**	45 ± 4.6	23 ± 1.2	67 ± 4.5	45.0	6.48
**2f**	24 ± 1.1	2.4 ± 0.7	11 ± 0.98	12.5	228
**2g**	0.47 ± 0.14	0.28 ± 0.07	0.95 ± 0.012	0.57	197
**2h**	82 ± 14	63 ± 8	>100	>81.7	>0.80
**2i**	>100	>100	>100	>100	~1.00
**3a**	1.1 ± 0.01	0.36 ± 0.10	1.3 ± 0.33	0.92	290
**3b**	3.8 ± 0.71	3.2 ± 1.1	5.7 ± 0.67	4.23	3325
**3c**	41 ± 2.5	38 ± 8.7	45 ± 11	41.3	0.91
**3d**	>100	>100	>100	>100	>0.02
**3e**	20 ± 1.3	19 ± 2.7	35 ± 4.2	24.7	8.03
**3f**	1.1 ± 0.084	1.6 ± 0.078	1.8 ± 0.050	1.50	32.0
**3g**	0.093 ± 0.020	0.18 ± 0.030	0.30 ± 0.047	0.19	123
**4**	3.0 ± 0.21	3.7 ± 2.0	5.3 ± 0.61	4.00	120
Melphalan	73 ± 15	37 ± 7.3	92 ± 7.8	67.3	0.80
Curcumin	70 ± 12	34 ± 6.8	32 ± 6.4	45.3	0.34

^a^ The CC_50_ values are the concentrations of the compounds required to kill 50% of the cells. ^b^ The potency-selectivity expression (PSE) figures of the compounds are the products of the reciprocal of the average CC_50_ value and the selectivity index (SI) figures times 100.

**Table 3:** ADME parameters of **2a**, **b**, **f**, **g**, **3a**, **b**, **f**, **g**, and **4**.

Compound	MW ^a^	LogP ^a^ (WLOGP)	H-Bond Acceptors	H-Bond Donors	Rotatable Bonds	TPSA ^a^	Violations
**2a**	604.69	4.95	4	0	7	74.76	2
**2b**	676.65	7.18	8	0	7	74.76	3
**2f**	844.90	5.02	12	0	15	148.6	5
**2g**	965.00	5.05	16	0	19	185.52	4
**3a**	618.72	5.34	4	0	8	74.76	3
**3b**	690.68	7.57	8	0	8	74.76	3
**3f**	858.93	5.41	12	0	16	148.6	5
**3g**	979.03	5.44	16	0	20	185.52	5
**4**	590.75	6.28	4	0	8	40.62	2
Ideal compound	<500	<5.00	<10	<5	<10	<140	

^a^ The abbreviations in some of the headings of each column refer to the molecular weight (MW), logarithm of the partition coefficient (Log P), hydrogen bond acceptor atoms, hydrogen bond donor atoms, and topological polar surface area (TPSA).

## Data Availability

Data contained within the article and Supplementary Materials.

## Supplementary Materials

The following supporting information can be downloaded at: https://www.mdpi.com/article/10.3390/medicines11010003/s1, Table S1: Search for correlations between various physicochemical parameters of the aryl substituents in **2a**–**g** and the CC_50_ values.; Table S2: Search for correlations between various physicochemical parameters of the aryl substituents in **3a**–**g** and the CC_50_ values and Table S3: Search for correlations between various physicochemical parameters of the aryl substituents in **2a**–**g** and **3a**–**c**, **e**–**g** and the SI values.
