# Supplementary material for: Dimeric 3,5-Bis(benzylidene)-4-piperidones: Tumor-Selective Cytotoxicity and Structure-Activity Relationships
Source: Medicines (Basel). 2024 Jan 11;11(1):3. doi: 10.3390/medicines11010003 (PMC10821124; doi:10.3390/medicines11010003)
Supplement: Supplementary file 1 [file medicines-11-00003-s001.zip › medicines-2562719-supplementary.pdf]

SUPPLEMENTAL SECTION

Dimeric 3,5-bis(benzylidene)-4-piperidones : Tumor-selective cytotoxicity and structure-activity relationships

Swagatika Das [a], *Praveen K. Roayapalley* [a,*], *Hiroshi Sakagami* [b], *Naoki Umemura* [c], *Dennis K. J. Gorecki* [a], *Mohammad Hossain* [d], *Masami Kawase* [e], *Umashankar Das* [a], *Jonathan R. Dimmock* [a]

[a] Drug Discovery and Development Research Cluster, University of Saskatchewan, Saskatoon, Saskatchewan S7N 5E5, Canada

[b] Meikai University Research Institute of Odontology, Sakado, Saitama 350-0283, Japan

[c] Department of Oral Biochemistry, Asahi University School of Dentistry, Gifu 501-0296, Japan

[d] School of Sciences, Indiana University Kokomo, Kokomo, IN 46904-9003, USA

[e] Faculty of Pharmaceutical Sciences, Matsuyama University, Matsuyama, Ehime 790-8578, Japan

*Corresponding author : E-mail : rpraveen.sp@usask.ca Tel : +13067154217

Table S1. Search for correlations between various physicochemical parameters of the aryl substituents in **2a-g** and the CC_{50} values.

Bioassay	Plot	σ	π	MR
HL-60	l	<0.05	<0.01	--
HL-60	sl	--	<0.01	--
HSC-2	l	<0.05	<0.01	--
HSC-2	sl	--	<0.05	--
HSC-3	l	<0.1	<0.05	--
HSC-3	sl	--	<0.05	--
HSC-4	l	<0.05	<0.01	--
HSC-4	sl	--	<0.05	--

Table S2. Search for correlations between various physicochemical parameters of the aryl substituents in **3a-g** and the CC_{50} values.

Bioassay	Plot	σ	π	MR
HL-60	l	<0.01	<0.05	--
HL-60	sl	<0.1	<0.01	--
HSC-2	l	<0.01	<0.05	--
HSC-2	sl	<0.1	<0.01	--
HSC-3	l	<0.01	<0.01	--
HSC-3	sl	<0.1	<0.01	--
HSC-4	l	<0.05	<0.05	--
HSC-4	sl	<0.1	>0.01	--

Please note the following comments which pertain to Tables S1 and S2.

1. The letters l and sl refer to the plots being linear and semilogarithmic.
2. Only relationships ($p<0.05$) or tendencies to a relationship ($p<0.1$) are placed in the tables.
3. No correlations are designated as --.
4. All the correlations are positive.

Table S3. Search for correlations between various physicochemical parameters of the aryl substituents in **2a-g** and **3a-c**,**e-g** and the SI values.

Compounds	Plot	σ	π	MR
**2a-g**	l	--	--	--
**2a-g**	sl	<0.05	<0.05	--
**3a-c,e-g**	l	--	--	--
**3a-c,e-g**	sl	--	--	--

Please note the following comments which pertain to Table S3.

1. The letters l and sl refer to the plots being linear or semilogarithmic.
2. The correlations noted are negative.
3. No correlations are designated --.
